# The rs1014290 Polymorphism of the SLC2A9 Gene Is Associated with Type 2 Diabetes Mellitus in Han Chinese

**DOI:** 10.1155/2011/527520

**Published:** 2011-04-14

**Authors:** Wan-Chun Liu, Chi-Chih Hung, Szu-Chia Chen, Ming-Yen Lin, Ling-I Chen, Daw-Yang Hwang, Jer-Ming Chang, Jer-Chia Tsai, Hung-Chun Chen, Shang-Jyh Hwang

**Affiliations:** ^1^Department of Internal Medicine, Kaohsiung Municipal Hsiaokang Hospital, Kaohsiung, Taiwan; ^2^Division of Nephrology, Department of Internal Medicine, Kaohsiung Medical University, Chung-Ho Memorial Hospital, 100 Tzyou First Road, Kaohsiung 807, Taiwan; ^3^Department of Internal Medicine, Faculty of Renal Care, College of Medicine, Kaohsiung Medical University, Kaohsiung, Taiwan

## Abstract

*Aims*. The *SLC2A9* gene encodes the glucose transporter 9, with the abilities of transporting both glucose and uric acid and is involved in the pancreatic glucose-stimulated insulin secretion. The single nucleotide polymorphisms (SNPs) of *SLC2A9* accounted for 5% variance of serum uric acid (UA). UA was identified as a risk factor for type 2 diabetes mellitus (DM). We investigated whether the *SLC2A9* gene variations are associated with type 2 DM in Han Chinese. *Methods*. Three common SNPs of the *SLC2A9*, rs1014290, rs2280205, and rs3733591, were genotyped in 1003 Han Chinese randomly selected from Kaohsiung, Taiwan. *Results*. The variant SNP rs1014290 is associated with decreased 0.12-fold risk of type 2 DM (*P* = .002). Per-copy increase in the minor C-allele results in 0.13 mmol/L (*P* = .037) and 10.03 *μ*mol/L (*P* = .016) decrease in serum glucose and UA, respectively. *Conclusions*. The SNP rs1014290 within the *SLC2A9* gene is associated with type 2 DM in Han Chinese.

## 1. Introduction

Type 2 diabetes mellitus (DM), characterized by insulin resistance and relative insulin deficiency, is a complex disease with contributions from both genetic and environmental factors. Serum uric acid (UA) was associated with insulin resistance and also a risk factor for development of type 2 DM [1–5]. Meta-analysis showed that the pooled relative risk of a 1 mg/dL increase in UA was 1.17 for incident type 2 DM [5]. In the animal model, elevated UA level may worsen insulin resistance by inhibition of the bioavailability of nitric oxide, which is one of the essential components of insulin-stimulated glucose uptake [6]. The genetic influences outweighed the environmental factors in the estimation of serum UA by 63–73% [7, 8], with total genetic variability accounted for 5% of UA variance [9]. The common variants of *SLC2A9* gene have a significant role in the UA level, which accounted for 5-6% and 1-2% variables in the female and male, respectively.

The *SLC2A9* gene encodes the solute carrier family 2, facilitated glucose transporter member 9 (GLUT9), initially identified by sequence similarity with members of the glucose transporter (Glut) family [10]. The function of GLUT9 is a high-capacity uric acid transporter in addition to a glucose transporter [11–14]. Furthermore, GLUT9 affected glucose-stimulated insulin secretion in the pancreatic *β* cell and can be upregulated in the diabetic mouse [15, 16]. 

Due to the GLUT9's dual capabilities of uric acid and glucose transportations, we proposed that the *SLC2A9* variations might be associated with type 2 DM. In this study, we investigated three common single nucleotide polymorphisms (SNP) of the SLC2A9 gene and their association with type 2 DM in the Han Chinese. 

## 2. Method

### 2.1. Study Participants

 A cross-sectional survey was performed from one million population of Kaohsiung County, Taiwan. Twelve villages were randomized selected from twenty-seven villages with a total of 1003 subjects sampled through multistage stratified approaches between April 2007 and January 2008. Height, body weight, abdominal girth, and blood pressure were measured at the screen day. All clinical information including underlying diseases, drug history, and social habits were accomplished by trained persons via a standard questionnaire according to the subject's statement. Aborigines and foreigners were excluded from this survey. The type 2 DM was diagnosed by the subject's statement and serum glucose above 7 mmol/L (126 mg/dL).

### 2.2. Genotype Analysis

 Three SNPs (rs2280205, rs1014290, and rs3733591) of the *SLC2A9* gene were selected for genotype analysis with minor allele frequency >20% in Han Chinese (according to HapMap data). These three SNPs were reported to be associated with uric-acid-related phenotypes, such as gout and tophus formation [13, 17]. DNA was collected from the peripheral blood leukocytes and genotyping was performed by the TaqMan SNP allelic discrimination assay with an ABI 7900HT Sequence Detection System [18].

### 2.3. Statistical Analysis

 The differences of clinical characteristics between sexes, diabetes, and genotypes were analyzed by Student's *t*-test, Pearson's Chi-square test, Fisher's exact test, and analysis of variance (ANOVA). The genotype and allele frequencies for Hardy-Weinberg equilibrium proportions were tested by Pearson's Chi-square test. Linear regression was used to investigate the changes in uric acid and serum glucose for per-copy increment in the minor allele. Binary logistic regression was used to evaluate the risk of type 2 DM between genotype groups. Statistical analyses were performed with SPSS version 18. *P* values less than  .05 were considered statistically significant. Subjects were analyzed by sex stratification to evaluate the gender influence of the *SLC2A9* on type 2 DM, since the *SLC2A9* has a female-predominant effect on UA level.

## 3. Result

 The minor allele frequencies of the investigated SNPs were 42.1% for rs1014290, 22.7% for rs2280205, and 34.1% for rs3733591. These genotype frequencies were similar to the Han Chinese of the Hapmap database and all were in agreement with the Hardy-Weinberg equilibrium. All three loci are not in linkage. The demographic and clinical characteristics of study participants stratified by sex and DM were shown in Table 1. In the sex stratification, female had lower body mass index (BMI), mean arterial pressure (MBP), uric acid, hemoglobin, triglyceride, estimated glomerular filtration rate (eGFR), and percentage of gout but higher high-density lipoprotein (HDL). In the DM stratification, the DM subgroup was older and has higher BMI, MBP, fasting serum glucose, uric acid, cholesterol, triglyceride, low-density lipoprotein (LDL), and higher percentages of hypertension and metabolic syndrome but lower HDL and eGFR than non-DM subgroup. The genotype frequencies of the SNP rs1014290 were lower in the female and the DM subgroup.

### 3.1. Genotype Analysis of Investigated SNPs

The rs1014290 showed different frequencies of type 2 DM between genotype groups (Table 2). Lower glucose (*P* = .006), uric acid (*P* = .002), and type 2 DM (*P* = .019) were found to be associated with the rs1014290 only in the female but not in the male. There were no differences in the other clinical characteristics between genotype groups. 

In all subjects, each copy of SNP rs1014290 minor C-allele decreased covariate adjusted glucose and uric acid level by 0.13 mmol/L (*P* = .037) and 10.03 *μ*mol/L (*P* = .006), respectively. In female subgroup, the decreased effect of minor C-allele on glucose and uric acid level was more predominant. Each copy increase of the minor C-allele resulted in 0.22 mmol/L decrease (*P* = .018) and 18.39 *μ*mol/L decrease (*P* < .001) in covariate adjusted glucose level and uric acid level, respectively. The significant effect of SNP rs1014290 on glucose and uric acid level decrease was not replicated in male participants (Table 3).

In all subjects, each copy of SNP rs1014290 minor C-allele decreased covariate adjusted glucose and uric acid level by 0.13 mmol/L (*P* = .037) and 10.03 *μ*mol/L (*P* = .006), respectively. In the female subgroup, this effect was more predominant with 0.22 mmol/L decrease (*P* = .018) and 18.39 *μ*mol/L decrease (*P* < .001) in covariate adjusted glucose level and uric acid level, respectively. This effect of SNP rs1014290 on glucose and uric acid level decrease was not found in the male participants (Table 3). 

For all subjects and sex subgroup, the CC group of SLC2A9 SNP rs1014290 is associated with decreased risk for prevalent type 2 DM either in unadjusted or in adjusted model (Table 4). In the total group, compared with the TT group, the CC group conferred covariate adjusted 0.12-fold decreased risk of prevalent type 2 DM (95% confidence interval (CI) = 0.03–0.45, *P* = .002). In the subgroup analysis, this effect still existed in both sexes with weaker association in the male than in the female (male : odds ratio (OR) = 0.09, 95% CI = 0.01–0.93, *P* = .043; female: OR = 0.13, 95% CI = 0.02–0.70, *P* = .018). In contrary to the rs1014290 polymorphism, the uric acid only exerts a significant effect on the prevalent type 2 DM in unadjusted model of total and female group (OR for 10 *μ*mol/L increase in UA level = 1.02, 95% CI = 1.00–1.05, *P* = .036; OR for 10 *μ*mol/L increase in UA level = 1.06, 95% CI = 1.02–1.09, *P* < .001, resp.). The significant results of the rs1014290 polymorphism on the prevalent type 2 DM in the adjusted model suggested that the association between type 2 DM and *SLC2A9* variation is independent from the uric acid effect.

In the remaining variants of rs2280205 and rs3733591, no significant effects on the glucose, UA, and type 2 DM were found (Data not shown).

## 4. Discussion

We showed that in Han Chinese, the *SLC2A9* SNP rs1014290 is relevant to the prevalent of type 2 DM and serum glucose level in addition to the uric acid level. SNP rs1014290 variation is a nucleotide transversion from T to C located in the intron 3 of *SLC2A9* gene. Its minor allele frequency in Asian is about 40%, which is higher than the 30% in Caucasian according to HapMap data. The minor C-allele of SNP rs1014290 was found to associate with lower UA level, higher fractional excretion of uric acid and lower risks for gout and nephrolithiasis [13, 19]. The effect on decreasing uric acid level of minor C-allele of SNP rs1014290 is greater in female than in male. The influence of the *SLC2A9* variation on glucose level is more predominant in the female, as well as uric acid showed in our study and previous reports [11–14]. In the total group, this female predominant effect on uric acid is powerful enough to make the insignificant unadjusted *P* value become significant in adjusted model. The *P* value changed from  .075 to  .002 when sex was added as covariate to the unadjusted model. In the Caucasian, the per copy of minor C-allele contributes to 45.2–50.6 *μ*mol/L (0.76–0.85 mg/dL) decrease of serum uric acid level in the female and 10.1–21.4 *μ*mol/L (0.17–0.36 mg/dL) decrease in the male [13]. The effect of minor C-allele on decreasing uric acid level seems to be weaker in Han Chinese. In our study, each copy of minor C-allele decreases uric acid by 19.35 *μ*mol/L and 5.05 *μ*mol/L in female and in male, respectively, and this effect is insignificant in male. Similar result was found in a recent study including 191 male Han Chinese from Taiwan, where no influences of SNP rs1014290 variation on uric acid level were found [17]. Our data suggested that the SNP rs1014290 variation in the *SLC2A9* is associated with serum uric acid level in the Han Chinese with female predominance.

 GLUT9 was classified as glucose and/or fructose transporter despite very low transport activities [20, 21]. Recently, the GLUT9 was considered as a uric acid transporter rather than glucose and/or fructose transporter [11–14]. However, our study showed that the SNP rs1014290 variation is associated with type 2 DM and serum glucose level (Tables 3 and 4). GLUT9 was found to be upregulated in liver and kidney tissue in diabetic mouse and affected the glucose-sensing insulin secretion in pancreatic *β* cell [15, 16]. Pancreatic *β* cell was responsible for detecting the change of blood glucose concentration and modulating insulin secretion, which consisted of a rapid first phase and the prolonged second phase. The first step for the pancreatic *β* cell to sense extracellular glucose concentration is the uptake of glucose by glucose transporter. GLUT9 might be responsible for this glucose uptake in the prolonged second phase of insulin secretion. [15] Thus, the minor C-allele of SNP rs1014290 might exert an influence on the second phase of insulin secretion and consequently altered serum glucose level and type 2 DM development. In our result, the association between SNP rs1014290 variation and the prevalence of type 2 DM is independent from the effect of uric acid. 

Uric acid was identified as a risk factor for the development of type 2 DM in other studies [1–5]. But our result was only significant in unadjusted model of total and female groups (Table 4). This might be due to the older age of our DM subjects than that of other study subjects. The mean age of our DM subjects is around 62.5 years which is older than other study subjects (around 40–60 years) [5]. This suggested that the age *per se* and the duration of DM might affect the association between UA and type 2 DM.

 The minor allele frequency of SNP rs2280205 and SNP rs3733591 in our study is similar with that reported in Han Chinese group in HapMap data. The association between uric acid level and variations of SNP rs2280205 and SNP rs3733591 were reported [11], but the result was not replicated in our Han Chinese subjects. This may be due to the minor allele frequencies of SNP rs2280205 and SNP rs3733591 which are different between European and Han Chinese.

 The limitations of this are that our study is lacking the accurate diagnosis of type 2 DM and only random glucose level was tested without the measurement of glycosylated hemoglobin. This might lead to the finding that the SNP rs1014290 variation is not associated with serum glucose level but associated with type 2 DM in the male group. This contradictory result could also derive from the confounding effect of antidiabetic agents use and the inconsistent treatment response to DM medication. All these could confound the interpretation of the genetic effects on type 2 DM risk.

In conclusion, the SNP rs1014290 variation of *SLC2A9* gene was associated with type 2 DM and serum glucose level in addition to uric acid level. The genetic effect on the association with type 2 DM is independent from the uric acid effect. This suggested that the *SLC2A9* gene may be a candidate genetic locus for the pathogenesis of type 2 DM.

## Figures and Tables

**Table 1:** Clinical characteristics stratified by sex and DM of enrolled subjects.

Stratification	Sex		*P* value	DM		*P* value
	Male	Female		Non-DM	DM	
Subject No.	484	519		923	80	
Age (years)	49.0 ± 0.8	47.6 ± 0.7	.236	47.1 ± 0.6	62.5 ± 1.3	<.001
Sex (male%)	—	—	—	48.6%	43.8%	.245
BMI (kg/m^2^)	24.7 ± 0.2	23.5 ± 0.2	<.001	23.9 ± 0.1	25.3 ± 0.6	.013
MBP (mmHg)	98.5 ± 1.0	92.0 ± 0.7	<.001	94.0 ± 0.5	107.9 ± 4.3	.002
Glucose (mmol/L)	4.85 ± 0.07	4.84 ± 0.07	.985	4.55 ± 0.02	5.99 ± 0.35	<.001
Uric Acid (*μ*mol/L)	410.4 ± 6.0	303.3 ± 6.0	<.001	350.9 ± 5.9	380.7 ± 11.9	.067
Hemoglobin (g/L)	150.8 ± 0.6	132.3 ± 0.6	<.001	141.1 ± 0.5	141.9 ± 1.5	.678
Cholesterol (mmol/L)	4.97 ± 0.04	5.08 ± 0.04	.066	5.01 ± 0.03	5.25 ± 0.12	.036
Triglyceride (mmol/L)	1.55 ± 0.06	1.24 ± 0.04	<.001	1.32 ± 0.03	2.21 ± 0.18	<.001
HDL (mmol/L)	1.41 ± 0.02	1.68 ± 0.02	<.001	1.57 ± 0.01	1.31 ± 0.04	<.001
LDL (mmol/L)	3.14 ± 0.04	3.10 ± 0.04	.513	3.10 ± 0.03	3.30 ± 0.11	.048
eGFR (mL/s)	1.38 ± 0.02	1.30 ± 0.02	<.001	1.36 ± 0.02	1.08 ± 0.05	<.001
Type 2 DM (%)	7.2%	8.7%	.401	—	—	—
Hypertension (%)	17.9%	14.7%	.169	14.0%	42.5%	<.001
Metabolic syndrome (%)	10.1%	11.8%	.235	7.5%	51.3%	<.001
Cardiovascular disease (%)	4.8%	5.4%	.382	4.9%	7.5%	.219
Cerebrovascular disease (%)	1.5%	0.8%	.233	1.0%	2.5%	.219
Gout (%)	10.4%	2.1%	<.001	5.9%	8.8%	.211
Minor allele frequency						
rs1014290	44.3%	40.1%	.011	43.3%	28.8%	<.001
rs2280205	22.5%	23.0%	.576	22.5%	25.0%	.266
rs3733591	34.6%	33.6%	.245	33.5%	40.6%	.179

Values are expressed as mean ± SEM or percentage.

The *P* values were determined from *t*-tests for continuous variables and from Pearson chi-square test, and fisher's exact test for categorical variables.

Abbreviations: BMI: body mass index, MBP: mean blood pressure, HDL: high-density lipoprotein, LDL: low-density lipoprotein, eGFR: estimated glomerular filatration rate, DM: diabetes mellitus.

**Table 2:** Clinical characteristics stratified by rs1014290 C/T polymorphism in both sex in the SLC2A9 gene.

	Male			*P* value	Female			*P* value
	TT	CT	CC		TT	CT	CC	

Subjects No.	147	245	92		177	268	74	
Age (years)	49.9 ± 1.5	48.1 ± 1.2	48.1 ± 1.9	.617	47.6 ± 1.3	47.6 ± 1.0	49.8 ± 1.8	.582
BMI (kg/m^2^)	24.8 ± 0.3	24.7 ± 0.4	24.2 ± 0.4	.651	23.8 ± 0.3	23.5 ± 0.3	23.2 ± 0.5	.625
MBP (mmHg)	101.5 ± 2.6	97.7 ± 0.9	94.3 ± 1.5	.032	92.8 ± 1.1	92.4 ± 0.9	90.6 ± 1.6	.580
Glucose (mmol/L)	4.92 ± 0.14	4.79 ± 0.09	4.64 ± 0.07	.298	5.17 ± 0.17	4.78 ± 0.07	4.56 ± 0.10	.006
Uric Acid (*μ*mol/L)	410.4 ± 6.0	410.4 ± 6.0	398.5 ± 11.9	.575	321.2 ± 6.0	303.3 ± 6.0	279.6 ± 6.0	.002
Hemoglobin (g/L)	151.9 ± 1.1	150.8 ± 0.8	149.0 ± 1.5	.269	133.4 ± 1.0	132.1 ± 0.9	130.2 ± 1.7	.243
Cholesterol (mmol/L)	5.01 ± 0.07	4.97 ± 0.06	4.89 ± 0.12	.622	5.16 ± 0.08	5.03 ± 0.06	5.08 ± 0.11	.405
Triglyceride (mmol/L)	1.65 ± 0.09	1.46 ± 0.06	1.52 ± 0.18	.306	1.32 ± 0.07	1.21 ± 0.05	1.32 ± 0.16	.435
HDL (mmol/L)	1.40 ± 0.03	1.44 ± 0.03	1.44 ± 0.04	.637	1.67 ± 0.03	1.66 ± 0.03	1.69 ± 0.05	.830
LDL (mmol/L)	3.14 ± 0.06	3.15 ± 0.06	3.05 ± 0.10	.630	3.17 ± 0.07	3.08 ± 0.05	3.06 ± 0.09	.517
eGFR (mL/s)	1.38 ± 0.04	1.40 ± 0.03	1.39 ± 0.05	.858	1.32 ± 0.03	1.28 ± 0.02	1.23 ± 0.04	.201
Type 2 DM (%)	9.5%	8.2%	1.1%	.036	13.0%	7.5%	2.7%	.018
Hypertension (%)	19.0%	17.2%	18.0%	.900	16.4%	14.3%	12.2%	.655
Metabolic syndrome (%)	11.6%	10.2%	7.6%	.614	13.0%	12.3%	6.8%	.346
Cardiovascular disease (%)	4.8%	4.1%	6.7%	.607	5.7%	4.9%	6.8%	.809
Cerebrovascular disease (%)	2.0%	1.2%	1.1%	.777	1.1%	0.8%	0.0%	.647
Gout (%)	15.1%	8.2%	9.0%	.088	3.4%	1.5%	1.4%	.356

Values are expressed as mean ± SEM or percentage.

The *P* values were determined from *t*-tests for continuous variables and from Pearson chi square test and fisher's exact test for categorical variables.

Abbreviations: as Table 1.

**Table 3:** Glucose and uric acid level differences for per-copy increase of minor allele for rs1014290 in the SLC2A9 gene.

	Glucose		Uric Acid	
	Unstandardized coefficient *β*	*P* value	Unstandardized coefficient *β*	*P* value

Total				
Unadjusted	−0.24	.001	−8.17	.080
Adjusted	−0.13	.037	−10.03	.006

Male				
Unadjusted	−0.13	.156	−5.05	.391
Adjusted	−0.03	.759	−2.35	.676

Female				
Unadjusted	−0.34	.002	−19.35	<.001
Adjusted	−0.22	.018	−18.39	<.001

Values are expressed as unstandardized coefficient.

The *P* values were determined from linear regression analysis.

Adjusted for age, eGFR, MBP, cholesterol, triglyceride, HDL, BMI, antidiabetic agents and sex in total group (sex was excluded in sex subgroup analysis) in glucose analysis.

Adjusted for age, eGFR, MBP, cholesterol, triglyceride, HDL, BMI, and sex in total group (sex was excluded in sex subgroup analysis) in uric acid analysis.

**Table 4:** Binary logistic regression analysis for type 2 DM risk.

	Unadjusted		Adjusted model 1		Adjusted model 2	
	OR (95% CI)	*P*	OR (95% CI)	*P*	OR (95% CI)	*P*

Total						
rs1014290 variation						
TT	1 (Reference)	—	1 (Reference)	—	1 (Reference)	—
CT	0.63 (0.39–1.01)	.056	0.66 (0.40–1.09)	.101	0.67 (0.39–1.13)	.136
CC	0.15 (0.05–0.49)	.002	0.15 (0.05–0.51)	.002	0.12 (0.03–0.45)	.002
Uric acid	1.02 (1.00–1.05)	.036	1.01 (0.98–1.04)	.389	0.99 (0.96–1.02)	.610

Male						
rs1014290 variation						
TT	1 (Reference)	—	1 (Reference)	—	1 (Reference)	—
CT	0.81 (0.39–1.67)	.564	0.85 (0.40–1.79)	.672	0.86 (0.37–1.97)	.719
CC	0.11 (0.01–0.86)	.036	0.12 (0.02–0.94)	.043	0.09 (0.01–0.93)	.043
Uric acid	1.01 (0.97–1.05)	.651	1.00 (0.96–1.04)	.944	0.98 (0.94–1.03)	.398

Female						
rs1014290 variation						
TT	1 (Reference)	—	1 (Reference)	—	1 (Reference)	—
CT	0.52 (0.27–0.98)	.044	0.53 (0.26–1.05)	.069	0.50 (0.24–1.05)	.066
CC	0.19 (0.04–0.81)	.025	0.17 (0.04–0.76)	.021	0.13 (0.02–0.70)	.018
Uric acid	1.06 (1.02–1.09)	.001	1.01 (0.97–1.05)	.533	0.99 (0.95–1.05)	.724

Values are expressed as odds ratio (95% confidence interval). For uric acid, odds ratio was expressed for per 10 *μ*mol/L increase in level.

The *P* values were determined from binary logistic regression analysis.

Model 1: adjusted for age, BMI, uric acid, the rs1014290 C/T polymorphism of SLC2A9 gene, and sex in total group (sex was excluded in sex subgroup analysis).

Model 2: adjusted for covariates of model 1, MBP, cholesterol, HDL, LDL, and triglyceride.

Abbreviations: as Table 1.
